# COVID‐19 outcomes among rheumatic disease patients in Kuwait: Data from the COVID‐19 Global Rheumatology Alliance (C19‐GRA) physician registry

**DOI:** 10.1111/1756-185X.14332

**Published:** 2022-05-11

**Authors:** Fatemah Abutiban, Khulood Saleh, Sawsan Hayat, Hoda Tarakmah, Adeeba Al‐Herz, Aqeel Ghanem

**Affiliations:** ^1^ Department of Medicine Jaber Alahmed Hospital Ministry of Health Kuwait City Kuwait; ^2^ Department of Medicine Farwaniyah Hospital Ministry of Health Kuwait City Kuwait; ^3^ Department of Medicine Mubarak Alkabeer Hospital Ministry of Health Kuwait City Kuwait; ^4^ Department of Medicine AlAmiri Hospital Ministry of Health Kuwait City Kuwait

**Keywords:** anti‐rheumatic drugs, COVID‐19, Global Rheumatology Alliance, Kuwait, rheumatic disease

## Abstract

**Purpose:**

We aimed to assess the characteristics of inflammatory rheumatic disease (IRD) patients in Kuwait diagnosed with COVID‐19 and the factors linked with hospitalization, complications, and mortality.

**Methods:**

Data of IRD patients from Kuwait diagnosed with COVID‐19 between March 2020 and March 2021, submitted to the COVID‐19 Global Rheumatology Alliance physician‐reported registry, were included in our analysis. Data on patients' age, gender, smoking, diagnosis, IRD activity, and other comorbidities were collected. Statistical Package for the Social Sciences (SPSS), version 25, was used for statistical analysis.

**Results:**

A total of 52 patients were included, with a mean age of 55 years (±14). The majority of patients were ≤65 years (77%), female (77%), non‐smokers (80.8%), and diagnosed with rheumatoid arthritis (67.0%). Of the included patients, 19.2%, 9.6%, and 7.7% reported having methotrexate monotherapy, antimalarials monotherapy, and interleukin‐6 inhibitors monotherapy immediately before COVID‐19, respectively. Most of the included patients (92.3%) were either in remission or had minimal/low disease activity, while others (7.7%) had moderate disease activity. Forty‐three patients (82.7%) were hospitalized, while 11 patients (25.6%) required ventilation (invasive or non‐invasive). Ten of the ventilated patients (90.9%) received glucocorticoids as part of the local protocol to treat severe COVID symptoms, and 4 patients (7.69%) died. The duration till symptom‐free ranged between 0 to 30 days, with a mean value of 10 days (±6.5).

**Conclusion:**

The current study provides timely real‐world evidence regarding characteristics and potential risk factors linked to poor COVID‐19‐related outcomes in the IRD population in Kuwait.

## 1 INTRODUCTION

There is insufficient reliable data to guide our knowledge of outcomes in patients with inflammatory rheumatic diseases (IRD) or those who are immunosuppressed after SARS‐CoV‐2 infection, leading to uncertainty about chronic disease treatment in such patients.^1^, ^2^, ^3^ Previous literature has highlighted the uncertainty if individuals with IRD fall into a susceptible, higher‐risk group for being infected with SARS‐CoV‐2 and have poor outcomes.^4^, ^5^, ^6^ Compared to people without IRD, IRD patients appear to have similar or slightly worse results.^5^, ^7^ However, crucial illness‐related confounding variables (eg, disease activity or therapies) have not been previously discussed.

COVID‐19 and its subsequent complications have been treated with medications typically used to treat IRD, raising issues regarding the influence of these therapies on SARS‐CoV‐2 infection outcomes.^8^, ^9^, ^10^ Previous literature had even suggested continuing immunomodulatory or immunosuppressive medication in order to control IRD activity, avoid the progression of the disease, and avoid joint/organ damage caused by chronic inflammation.^11^ Even during a pandemic, the withdrawal of effective medicines should be supported by scientific data.

In March 2020, a worldwide network of rheumatologists, data scientists, as well as patients, created a COVID‐19 physician‐reported case registry to collect more comprehensive data related to IRD patients infected with SARS‐CoV‐2.^12^, ^13^ Analyzing the collected data showed that older age, as well as comorbidities, were linked to hospitalization and severe COVID‐19 outcome compared to the findings in the general population.^4^, ^14^, ^15^

The current study aimed to assess the clinical characteristics of IRD patients in Kuwait diagnosed with COVID‐19 from the data submitted to the COVID‐19 Global Rheumatology Alliance (C19‐GRA) physician‐reported registry. Moreover, we investigated the factors linked with hospitalization, complications, and mortality among these patients.

## 2 METHODS

### 2.1 Study population and data source

In the current study, we included patients from Kuwait who entered the registry as of March 2020. The detailed C19‐GRA physician‐reported registry has been previously described.^12^, ^16^, ^17^ The data collected was multicenter, with Jaber Alahmed Alsabah Hospital being the source of most cases as the major COVID‐19 center in Kuwait.

The included patients were IRD patients with a COVID‐19 diagnosis. The diagnosis of COVID‐19 was reported by the physicians, whether it was diagnosed by polymerase chain reaction (PCR) test, metagenomic analysis, computed tomography imaging, laboratory investigations, or preliminary clinical diagnosis based on the clinical manifestations. Data on patients' age, gender, the status of smoking, medications prior to COVID‐19 diagnosis, IRD activity, and other comorbidities were captured. Moreover, we collected laboratory findings and COVID‐19‐related data in terms of the time of diagnosis, clinical manifestations, treatment, admission to the hospital, and the maximum level of care received.

### 2.2 Medications prior to COVID‐19

The medications before COVID‐19 diagnosis were categorized as follows:

1. conventional synthetic disease‐modifying anti‐rheumatic drugs (csDMARDs): antimalarials (hydroxychloroquine, chloroquine), azathioprine, cyclophosphamide, cyclosporine, leflunomide, methotrexate, mycophenolate mofetil/mycophenolic acid, sulfasalazine, and tacrolimus
2. biologic DMARDs (bDMARDs): abatacept, belimumab, CD‐20 inhibitors, interleukin (IL)‐1 inhibitors, IL‐6 inhibitors, IL‐12/IL‐23 inhibitors, IL‐17 inhibitors, tumor necrosis factor inhibitors (anti‐TNF), and
3. targeted synthetic DMARDs (tsDMARDs), namely Janus kinase (JAK) inhibitors.

The physicians reported the duration from the onset of symptoms either until the resolution of the symptoms or death.

### 2.3 Statistical analysis

For statistical analysis, Statistical Package for the Social Sciences (SPSS) for Windows, version 25, was used. Continuous variables are reported as mean and standard deviation (SD) for normally distributed data or median and interquartile range (IQR) for non‐normally distributed data. While dichotomous data are reported as frequency and percentage (%).

## 3 RESULTS

### 3.1 Demographic and clinical characteristics at the time of hospitalization

As of March 2021, a total of 52 Kuwaiti patients were included in the C19‐GRA physician‐reported registry. The mean age of the included patients was 55 years (SD = 14). Most of them were aged ≤65 years (n = 40, 77%), female (n = 40, 77%), Arab (n = 49, 94.2%), and never‐smokers (n = 42, 80.8%). The most common primary rheumatology diagnosis was rheumatoid arthritis (n = 35, 67.0%), followed by systemic lupus erythematosus (n = 6, 12.0%). Twenty‐eight patients (54%) were hypertensive, while 19 patients (37%) had diabetes. Interstitial lung disease was reported in 6 patients (12%), and obstructive lung disease was reported in 4 patients (8%).

**FIGURE 1:**
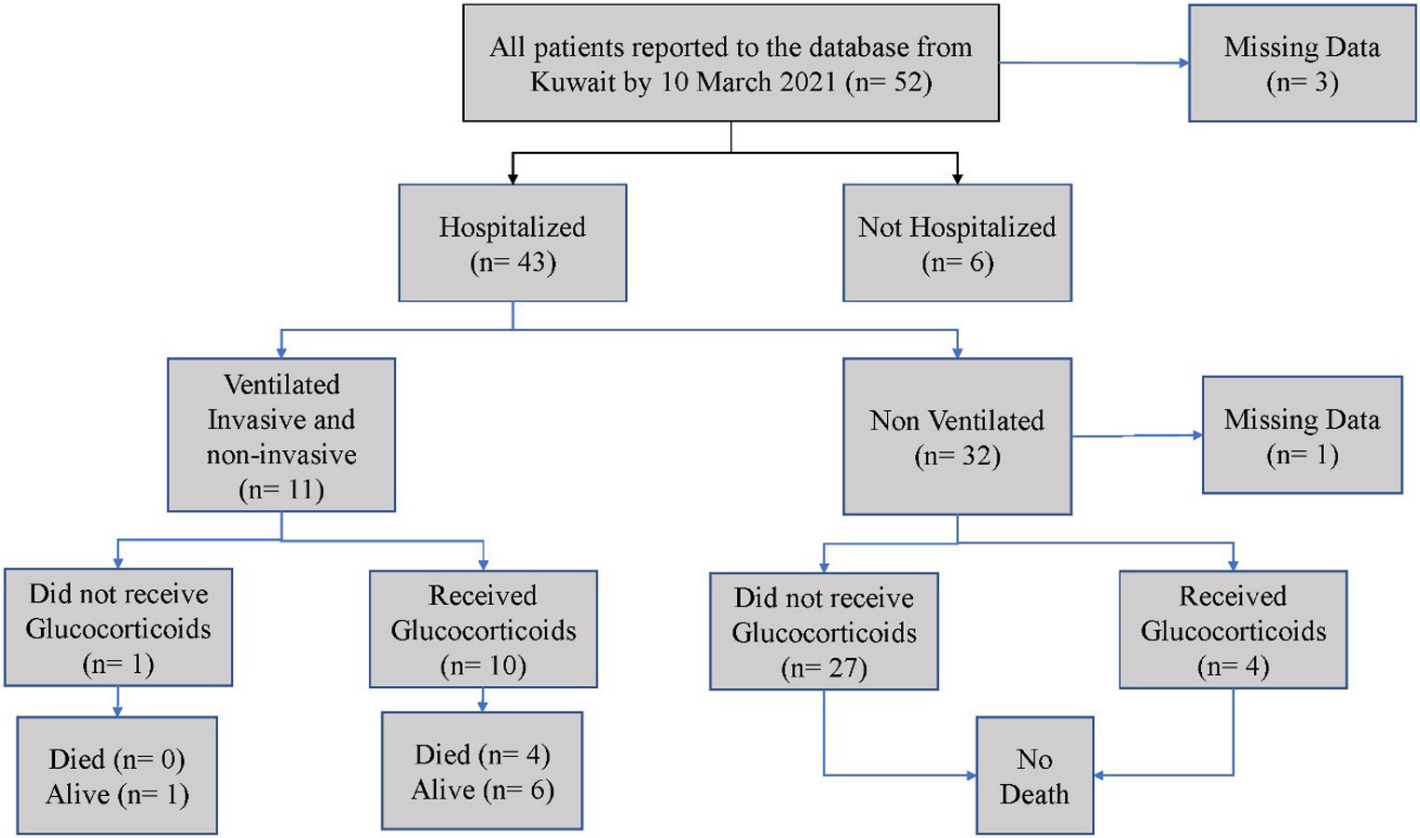
Flow diagram of the included patients

Ten patients (19.2%) reported having methotrexate monotherapy before COVID‐19 onset, while antimalarials monotherapy and IL‐6 inhibitors monotherapy were reported in 5 patients (9.6%) and 4 patients (7.7%), respectively, Table S1. Of the participants, 44 patients (85%) reported no use of glucocorticoids at the time of COVID‐19 symptom onset. The rheumatic disease activity was classified into remission, minimal/low disease activity, and moderate disease activity. Most of the included patients were either in remission or had minimal/low disease activity (n = 48, 92.3%), while only 4 patients (7.7%) had moderate disease activity. Of the included patients, only 2 (3.85%) received the COVID‐19 vaccine, 12 patients did not, while vaccination status was unknown for the majority of patients (n = 38; 73.08%). The demographic and clinical characteristics of the study participants are shown in Table 1. Figure 1 shows the flowchart of the included patients. The most common COVID‐19 symptoms at the time of presentation were cough (60%), fever (54%), and shortness of breath (48%), as shown in Figure S1. The majority of the included patients (n = 40, 76.9%) showed an absence of leukopenia (defined as white blood cells [WBCs] <5000/mm^3^). The laboratory investigations for the included patients are shown in Table S2.

**TABLE 1:** Patient demographic and clinical characteristics (N = 52)

Characteristic	Study cohort
Age	55 ± 14
Aged ≤65 y	40 (77%)
Aged >65 y	12 (23%)
Gender	
Female	40 (77%)
Male	12 (23%)
Race/ethnic origin	
Arab	49 (94.2%)
Non‐Arab	3 (5.8%)
Smoking status	
Former smoker	1 (1.9%)
Never smoked	42 (80.8%)
Unknown smoking status	9 (17.3%)
Primary rheumatology diagnosis	
Rheumatoid arthritis	35 (67.0%)
Systemic lupus erythematosus	6 (12.0%)
Behçet's	2 (3.8%)
Inflammatory myopathy	2 (3.8%)
Sarcoidosis	2 (3.8%)
Antineutrophil cytoplasmic antibody‐associated vasculitis	1 (1.9%)
Autoinflammatory syndrome (including tumor necrosis factor‐associated periodic syndrome, cryopyrin‐associated periodic syndrome, familial Mediterranean fever)	1 (1.9%)
Axial spondylarthritis (including ankylosing spondylitis)	1 (1.9%)
Immunoglobulin G4‐related disease	1 (1.9%)
Mixed connective tissue disease	1 (1.9%)
Psoriatic arthritis	1 (1.9%)
Immune‐modulating medications immediately before COVID‐19 onset	
Conventional synthetic disease‐modifying antirheumatic drugs (csDMARDs) monotherapy	25 (48.1%)
Biologic DMARDs (bDMARDs) monotherapy	12 (23.1%)
Targeted synthetic DMARDs (tsDMARDs) monotherapy	2 (3.8%)
csDMARDs plus bDMARDs	7 (13.5%)
csDMARDs plus tsDMARDs	3 (5.8%)
Azathioprine / 6‐mercaptopurine plus colchicine	1 (1.9%)
None	2 (3.8%)
Glucocorticoids at time of COVID‐19 symptom onset	
Yes	7 (13%)
No	44 (85%)
Unknown	1 (1.9%)
Rheumatic activity	
Remission	26 (50%)
Minimal or low disease activity	22 (42%)
Moderate disease activity	4 (7.7%)
Comorbidities	
Hypertension	28 (54%)
Diabetes	19 (37%)
Interstitial lung disease	6 (12%)
Obstructive lung disease	4 (8%)
Chronic renal insufficiency or end‐stage renal disease	3 (6%)
Obesity, body mass index ≥ 30	2 (4%)
Cardiovascular disease	2 (4%)
Others^a^	8 (15%)

Note

Continuous data are reported as mean ± SD; dichotomous data are reported as number and percentage (%).

^a^ Includes other lung diseases, morbid obesity (BMI ≥ 40), cardiovascular disease (coronary artery disease, congestive heart failure), cerebrovascular disease, pulmonary hypertension, cancer, organ transplant recipient, immunoreactions, inflammatory bowel disease, liver disease, chronic neurological or neuromuscular disease, trisomy 21, psychiatric condition (eg, schizophrenia, bipolar disorder), macrophage activation syndrome (prior to COVID‐19 diagnosis), psoriasis, pregnancy, post‐partum (<6 weeks), or unknown.

Table 2 shows the clinical characteristics of the included patients at the time of hospitalization. Of the included patients, 43 (82.7%) were hospitalized, and 6 (11.5%) were not hospitalized. Only 7 patients (13%) reported using glucocorticoids at the time of COVID‐19 symptom onset. Nineteen patients (44.2%) did not require supplemental oxygen, while 13 patients (30.2%) required supplemental oxygen. Eleven patients (25.6%) required ventilation, either invasive or non‐invasive. Of the 11 ventilated patients, 10 patients received glucocorticoids as part of the local protocol to treat severe COVID symptoms, and 4 patients (7.69%) died. In contrast, none of the non‐ventilated patients have died. Adult respiratory distress syndrome was reported in 8 patients (15%), while sepsis was reported in 4 patients (7.7%). Regarding COVID‐19 management, only supportive care was used for 25 patients (48.1%), and glucocorticoids were administered to 18 patients (34.6%).

**TABLE 2:** Patients' clinical characteristics during hospitalization (N = 52)

Characteristic	Study cohort
Hospitalization	
Yes	43 (82.7%)
No	6 (11.5%)
Glucocorticoids at time of COVID‐19 symptom onset	
Yes	7 (13%)
No	44 (85%)
Duration till symptoms free (n = 33)	
Mean (±SD), d	10 (±6.5)
Range	0‐30
Maximum care level during hospitalization (n = 43)	
Did not require supplemental oxygen	19 (44.2%)
Required supplemental oxygen	13 (30.2%)
Required non‐invasive ventilation or high flow oxygen devices	3 (7.0%)
Required invasive mechanical ventilation or extracorporeal membrane oxygenation	8 (18.6%)
Complications	
No known complications	44 (85%)
Adult respiratory distress syndrome	8 (15%)
Sepsis	4 (7.7%)
Secondary infection	1 (1.9%)
Cytokine storm	1 (1.9%)
Other serious complications^a^	5 (9.6%)
Death	
Yes	4 (7.69%)
COVID‐19 treatment	
Supportive care only	25 (48.1%)
Glucocorticoids	18 (34.6%)
Lopinavir /ritonavir	2 (4.0%)
Lopinavir /ritonavir and glucocorticoids	1 (1.9%)
Lopinavir /ritonavir and glucocorticoids and other	1 (1.9%)
Meronem and glucocorticoids	1 (1.9%)
Remdesivir and glucocorticoids	1 (1.9%)
Antimalarials	1 (1.9%)
Interleukin‐1b inhibitors	1 (1.9%)
Colchicine	1 (1.9%)

Note

Continuous data are reported as mean ± SD; dichotomous data are reported as number and percentage (%). NB: hospitalization status is missing for 3 patients; Glucocorticoids status at time of COVID‐19 symptom onset is unknown in 1 patient.

^a^ Includes kidney failure/injury required dialysis, kidney failure/injury on dialysis, or pneumothorax.

### 3.2 IRD patients with COVID‐19 stratified by ventilation status

Based on the ventilation status, IRD patients diagnosed with COVID‐19 were classified into 2 groups: non‐ventilated and ventilated patients. Most of the 32 non‐ventilated patients were ≤65 years (n = 24, 75.0%), female (n = 26, 81.25%), Arab (n = 30, 93.75%), never smoked (n = 27, 84.38%), non‐diabetic (n = 21, 65.63%), hypertensive (n = 18, 56.25%), and did not receive glucocorticoids at time of COVID‐19 symptom onset (n = 28, 87.50%). Moreover, most of the 11 ventilated patients were ≤65 years (n = 8), female (n = 10), Arab (n = 11), never smoked (n = 10), non‐diabetic (n = 6), hypertensive (n = 7), and did not receive glucocorticoids at time of COVID‐19 symptom onset (n = 9) (Table 3).

**TABLE 3:** Demographic and disease characteristics of individuals with rheumatic disease diagnosed with COVID‐19 stratified by the ventilation status

	Non‐ventilated		Ventilated	
	Frequency	Percentage (%)	Frequency	Percentage (%)
Age				
≤65 y	24	75.00	8	72.73
>65 y	8	25.00	3	27.27
Gender				
Female	26	81.25	10	90.91
Male	6	18.75	1	9.09
Race/ethnic origin				
Non‐Arab	2	6.25	0	0.00
Arab	30	93.75	11	100.00
Smoking status				
Former smoker	0	0.00	0	0.00
Never smoked	27	84.38	10	90.91
Unknown	5	15.63	1	9.09
Glucocorticoids at time of COVID‐19 symptom onset				
No	28	87.50	9	81.82
Yes	4	12.50	2	18.18
Methotrexate monotherapy				
No	25	78.13	11	100.00
Yes	6	18.75	0	0.00
Missing	1	3.13	0	0.00
Conventional synthetic disease‐modifying antirheumatic drugs (csDMARDs) (other than methotrexate)				
No	24	75.00	5	45.45
Yes	7	21.88	6	54.55
Missing	1	3.13	0	0.00
Methotrexate plus other csDMARDs				
No	28	87.50	11	100.00
Yes	3	9.38	0	0.00
Missing	1	3.13	0	0.00
Biologic DMARDs (bDMARDs) / targeted synthetic DMARDs (tsDMARDs) monotherapy				
No	22	68.75	6	54.55
Yes	9	28.13	5	45.45
Missing	1	3.13	0	0.00
bDMARDs/tsDMARDs plus methotrexate				
No	25	78.13	11	100.00
Yes	5	15.63	0	0.00
Missing	2	6.25	0	0.00
Interstitial lung disease				
No	28	87.50	10	90.91
Yes	4	12.50	1	9.09
Obstructive lung disease				
No	30	93.75	9	81.82
Yes	2	6.25	2	18.18
Diabetes				
No	21	65.63	6	54.55
Yes	11	34.38	5	45.45
Hypertension				
No	14	43.75	4	36.36
Yes	18	56.25	7	63.64

Note

NB: ventilation status is missing in 9 patients; dichotomous data are reported as number and percentage (%).

Among the non‐ventilated patients, methotrexate monotherapy, csDMARDs (other than methotrexate), and biologics monotherapy were reported in 6 patients (18.75%), 7 patients (21.88%), and 9 patients (28.13%), respectively. Methotrexate plus other csDMARDs and biologics plus methotrexate combinations were reported in 3 patients (9.38%) and 5 patients (15.63%), respectively. On the other hand, methotrexate monotherapy, as well as methotrexate plus other csDMARDs and biologics plus methotrexate combinations, were reported in none of the ventilated patients. csDMARDs (other than methotrexate) and biologics monotherapy were reported in 6 patients (54.55%) and 5 patients (45.45%), respectively.

### 3.3 IRD patients with COVID‐19 stratified by COVID‐19 complications

The demographic characteristics and the immune‐modulating medications stratified by COVID‐19 complications are shown in Table 4. The majority of IRD patients diagnosed with COVID‐19 reported no known complications (n = 44). Of them, 32 patients were female, 34 patients aged ≤65 years, and 39 patients did not receive glucocorticoids at COVID‐19 symptom onset. COVID‐19‐related complications were reported only in 8 patients. Out of the 8 patients (15.4%) who experienced COVID‐19 complications, 6 were not on glucocorticoids (including prednisone, methylprednisolone) at the time of COVID‐19 symptom onset. Among the patients with COVID‐19‐related complications, methotrexate monotherapy, csDMARDs (other than methotrexate), and biologics monotherapy were reported in 10 patients (22.7%), 11 patients (25.0%), and 12 patients (27.3%), respectively. Methotrexate plus other csDMARDs and biologics plus methotrexate combinations were reported in 3 patients (6.8%) and 5 patients (11.4%), respectively. On the other hand, methotrexate monotherapy, as well as methotrexate plus other csDMARDs and biologics plus methotrexate combinations, were reported in none of the patients with no known complications. csDMARDs (other than methotrexate) and biologics monotherapy were reported in half of them (n = 4, 50.0%).

**TABLE 4:** Demographic and disease characteristics of rheumatic disease patients diagnosed with COVID‐19 stratified by the COVID‐19 complications

	No COVID‐19 complications		COVID‐19 complications	
	Frequency	Percentage (%)	Frequency	Percentage (%)
Age				
≤65 y	34	77.3	6	75.0
>65 y	10	22.7	2	25.0
Gender				
Female	32	72.7	8	100.0
Male	12	27.3	0	0.0
Race/ethnic origin				
Non‐Arab	3	6.8	0	0.0
Arab	41	93.2	8	100.0
Smoking status				
Former smoker	1	2.3	0	0.0
Never smoked	35	79.5	7	87.5
Unknown	8	18.2	1	12.5
Glucocorticoids at time of COVID‐19 symptom onset				
No	39	88.6	6	75.0
Yes	5	11.4	2	25.0
Methotrexate monotherapy				
No	32	72.7	8	100.0
Yes	10	22.7	0	0.0
Missing	2	4.5	0	0.0
Conventional synthetic disease‐modifying antirheumatic drugs (csDMARDs) (other than methotrexate)				
No	31	70.5	4	50.0
Yes	11	25.0	4	50.0
Missing	2	4.5	0	0.0
Methotrexate plus other csDMARDs				
No	39	88.6	8	100.0
Yes	3	6.8	0	0.0
Missing	2	4.5	0	0.0
Biologic DMARDs (bDMARDs) / targeted synthetic DMARDs (tsDMARDs) monotherapy				
No	30	68.2	4	50.0
Yes	12	27.3	4	50.0
Missing	2	4.5	0	0.0
bDMARDs/tsDMARDs plus methotrexate				
No	36	81.8	8	100.0
Yes	5	11.4	0	0.0
Missing	3	6.8	0	0.0
Interstitial lung disease				
No	39	88.6	7	87.5
Yes	5	11.4	1	12.5
Obstructive lung disease				
No	42	95.5	6	75.0
Yes	2	4.5	2	25.0
Diabetes				
No	30	68.2	3	37.5
Yes	14	31.8	5	62.5
Hypertension				
No	21	47.7	3	37.5
Yes	23	52.3	5	62.5

Note

Dichotomous data are reported as number and percentage (%).

### 3.4 IRD patients with COVID‐19 stratified by COVID‐19‐related mortality

Of the 52 included patients, only 4 patients (7.69%) died. The 4 patients were females aged ≤65 years. None of the patients who received glucocorticoids (including prednisone, methylprednisolone) at COVID‐19 symptom onset have died. Table 5 shows the demographic characteristics and the immune‐modulating medications stratified by COVID‐19 mortality. Among the patients who lived, methotrexate monotherapy, csDMARDs (other than methotrexate), and biologics monotherapy were reported in 10 patients (20.8%), 13 patients (27.1%), and 14 patients (29.2%), respectively. Methotrexate plus other csDMARDs and biologics plus methotrexate combinations were reported in 3 patients (6.2%) and 5 patients (10.4%), respectively. On the other hand, methotrexate monotherapy, as well as methotrexate plus other csDMARDs and biologics plus methotrexate combinations, were reported in none of the patients who died. Of the 4 deceased patients, mycophenolate mofetil/mycophenolic acid monotherapy was reported in only 1 patient, while CD‐20 inhibitors (rituximab within the last 12 months) were reported in 3 patients.

**TABLE 5:** Demographic and disease characteristics of rheumatic disease patients diagnosed with COVID‐19 stratified by COVID‐19 mortality

	Alive	Deceased
	Frequency (%)	Frequency (%)
Age		
≤65 y	36.0 (75.0%)	4.0 (100.0%)
>65 y	12.0 (25.0%)	0.0 (0.0%)
Gender		
Female	36.0 (75.0%)	4.0 (100.0%)
Male	12.0 (25.0%)	0.0 (0.0%)
Race/ethnic origin		
Non‐Arab	3.0 (6.2%)	0.0 (0.0%)
Arab	45.0 (93.8%)	4.0 (100.0%)
Smoking status		
Former smoker	1.0 (2.1%)	0.0 (0.0%)
Never smoked	39.0 (81.2%)	3.0 (75.0%)
Unknown	8.0 (16.7%)	1.0 (25.0%)
Glucocorticoids at time of COVID‐19 symptom onset		
No	42.0 (87.5%)	3.0 (75.0%)
Yes	6.0 (12.5%)	1.0 (25.0%)
Methotrexate monotherapy		
No	36.0 (75.0%)	4.0 (100.0%)
Yes	10.0 (20.8%)	0.0 (0.0%)
Missing	2.0 (4.2%)	0.0 (0.0%)
Conventional synthetic disease‐modifying antirheumatic drugs (csDMARDs) (other than methotrexate)		
No	33.0 (68.8%)	2.0 (50.0%)
Yes	13.0 (27.1%)	2.0 (50.0%)
Missing	2.0 (4.2%)	0.0 (0.0%)
Methotrexate plus other csDMARDs		
No	43.0 (89.6%)	4.0 (100.0%)
Yes	3.0 (6.2%)	0.0 (0.0%)
Missing	2.0 (4.2%)	0.0 (0.0%)
Biologic DMARDs (bDMARDs) / targeted synthetic DMARDs (tsDMARDs) monotherapy		
No	32.0 (66.7%)	2.0 (50.0%)
Yes	14.0 (29.2%)	2.0 (50.0%)
Missing	2.0 (4.2%)	0.0 (0.0%)
bDMARDs/tsDMARDs plus methotrexate		
No	40.0 (83.3%)	4.0 (100.0%)
Yes	5.0 (10.4%)	0.0 (0.0%)
Missing	3.0 (6.2%)	0.0 (0.0%)
Interstitial lung disease		
No	42.0 (87.5%)	4.0 (100.0%)
Yes	6.0 (12.5%)	0.0 (0.0%)
Obstructive lung disease		
No	44.0 (91.7%)	4.0 (100.0%)
Yes	4.0 (8.3%)	0.0 (0.0%)
Diabetes		
No	30.0 (62.5%)	3.0 (75.0%)
Yes	18.0 (37.5%)	1.0 (25.0%)
Hypertension		
No	21.0 (43.8%)	3.0 (75.0%)
Yes	27.0 (56.2%)	1.0 (25.0%)

### 3.5 Days of hospitalization

The duration till symptom‐free ranged between 0 to 30 days, with a mean value of 10 days (SD = 6.5). The detailed descriptive analysis of the days of hospitalization of the included patients is shown in Table S3.

## 4 DISCUSSION

The COVID‐19 pandemic undoubtedly influences the therapeutic approach to rheumatic diseases, whose infectious risk is considerably higher than the general population due to an overall immune system impairment characteristic of autoimmune diseases associated with the iatrogenic effect of corticosteroids as well as immunosuppressive drugs. Notably, numerous rheumatic medications, such as hydroxychloroquine, JAK, and IL‐6 inhibitors, are being investigated to prevent and/or manage COVID‐19 and its consequences.^8^, ^9^, ^10^ A worldwide network of rheumatologists, scientists, and patients created a physician‐reported case registry of patients with IRD confirmed with COVID‐19 diagnosis to fill this knowledge shortfall.^16^, ^17^

Kuwait's population is 4.67 million people as of 2021, with 1.85 million Kuwaitis and 2.8 million foreigners from more than 100 countries. Between March 2020 and March 2021, 230 596 COVID‐19 cases have been reported by the Ministry of Health of Kuwait.

Earlier, a prevalence phase of a study on data of patients with rheumatic diseases conducted by Al‐Awadhi et al on adult Kuwaitis^38^ showed that 2057 people were classified as “sufferers”, with a prevalence of musculoskeletal (MSK) pain of 26.8%. Male‐to‐female ratio was 1:1.9, and the mean age was higher in men than in women (47.5 years vs 44.4 years). A follow‐up study^39^ on the participants who had no MSK pain reported a new onset of MSK pain, with a prevalence of 6.6%. Of the 220 respondents, rheumatic conditions were reported in 29 patients (18 female and 11 male), with a male‐to‐female ratio of 1:1.6. The most frequent rheumatic condition was soft‐tissue rheumatism (n = 17).

Most of our study participants were female. This is consistent with the predominance of autoimmune diseases in females. Similar findings were reported in previous studies that assessed the impact of the COVID‐19 pandemic on IRD patients.^18^, ^19^ However, some literature reported a relative male predominance among IRD patients with severe SARS‐CoV‐2 infection.^20^, ^21^

As reported by the Jaber Hospital electronic medical registry, the total number of hospitalized patients with COVID‐19 in the same time period as our data collection was 13 825. A recent comparative study^22^ revealed that, compared with matched comparators, IRD patients had a higher risk of hospitalization (relative risk [RR] = 1.14) and intensive care unit (ICU) admission (RR = 1.32), but not mechanical ventilation or death (RR = 1.05 and 1.08). The risks were reduced when the model was broadened to include comorbidities as well as healthcare utilization.

Of the included patients, 82.7% were hospitalized, 25.6% required either invasive or non‐invasive ventilation, 15.4% had complications, and 4 female patients died. These findings reflect the increased rate of worse COVID‐19‐related outcomes, requiring ventilation, and death among females. Previous literature has reported contrary results. In the report by Hasseli et al, a total of 104 patients (63 female and 40 male) with IRD diagnosed with COVID‐19 were included. The authors documented an overall hospitalization rate of 32%; the proportion of male patients who required hospitalization was higher, even though both genders were roughly evenly represented. In their study, out of the hospitalized patients, 39% required either non‐invasive or invasive ventilation, and death was documented for 6 patients (3 female and 3 male).^20^

A previous comparative cohort study^19^ conducted on 52 IRD patients and 104 non‐rheumatic disease comparators showed a lower hospitalization rate among IRD patients diagnosed with COVID‐19 (n = 23, 44%). This percentage was similar to the proportion of hospitalized patients from the non‐rheumatic disease group (40%, *P* = .50). In their study, ICU admission and mechanical ventilation were required for 11 IRD patients (48%) compared with 7 (18%) non‐rheumatic disease comparators (odds ratio = 3.11, 95% confidence interval = 1.07 to 9.05), and the mortality rate was comparable between the 2 groups (6% of IRD patients vs 4% of non‐rheumatic disease comparators, *P* = .69).

Another prospective case series was conducted by Haberman et al^23^ involving patients with immune‐mediated inflammatory diseases. When proven or strongly suspected COVID‐19 infection emerged, the included patients received anti‐cytokine biologics monotherapy, immunomodulatory medications, or both. Fourteen patients (16%) were hospitalized. Compared with the hospitalized patients, the ambulatory patients (for whom hospitalization was not warranted) showed a higher percentage of being on biologics or JAK inhibitors at baseline (76% vs 50%). The overall hospitalization rate among individuals who had been on these therapies for a long time was 11%, and the multivariate analysis showed that patients with immune‐mediated inflammatory disorders who needed hospitalization used more oral glucocorticoids (29% vs 6%), hydroxychloroquine (21% vs 7%), and methotrexate (43% vs 15%) than the ambulatory patients. When their analysis was limited to individuals with proven SARS‐Cov‐2 infection based on PCR testing, these findings remained consistent, and of the 14 hospitalized patients, 1 patient died while the other patient had high levels of IL‐6 and required mechanical ventilation. None of the 2 patients received long‐term biologic therapy. Gianfrancesco et al (2020)^14^ showed that older age and comorbidities (such as diabetes mellitus, hypertension, cardiovascular disorders, etc) were associated with a higher risk of COVID‐19 hospitalization. This is supported by previous literature.^24^, ^25^, ^26^ As regards JAK inhibitors, Sparks et al (2021)^27^ documented that when compared to RA patients who used anti‐TNF therapies, RA patients who received rituximab or JAK inhibitors at the time of COVID‐19 infection were more likely to have poor COVID‐19 outcomes that ranged from hospitalization to death. The majority of patients with IRD are treated regularly with glucocorticoids, csDMARDs, and b/tsDMARDs. Some of the therapies used to manage IRD patients have been suggested to be useful in treating SARS‐CoV‐2 infection, while others may have negative side effects comparable to those seen in rituximab‐treated patients.^28^ Glucocorticoids, in particular, raise the risk of severe infection in a dose‐dependent way. Data from the C19‐GRA registry showed that a daily dose of glucocorticoids of ≥10 mg is linked to a greater risk of hospitalization.^14^ The use of DMARDs has been linked to the development of infectious problems; the majority of these infections are bacterial, although some viral infections, such as herpes zoster, can affect the course of numerous anti‐rheumatic treatments.^29^, ^30^

Gianfrancesco et al^14^ documented that high prednisone dosages (more than 10 mg/d) were linked to a higher risk of COVID‐19 hospitalization, while no link between previous NSAID or antimalarial usage and COVID‐19 hospitalization was found. Moreover, the authors stated that biologic or tsDMARDs monotherapy was linked to decreased hospitalization risk, primarily driven by anti‐TNF therapies. Previously, Richter et al (2016)^31^ documented that TNF inhibitors are linked to a higher risk of severe infections in the early stages of treatment, but as they become much more effective, the risk reduces due to improved functional ability and reduced glucocorticoids usage. Interestingly, a recent study conducted by Izadi et al (2021)^32^ on 6077 patients from 74 countries showed that, when compared to other frequently prescribed immunomodulatory management regimens, TNF inhibitor monotherapy was linked with a reduced risk of unfavorable COVID‐19 outcomes in individuals with immune‐mediated inflammatory diseases.

None of the patients who received glucocorticoids (including prednisone and methylprednisolone) at COVID‐19 symptom onset have died in the current study. Previous literature has documented that mycophenolate mofetil and rituximab were significantly linked with worse outcomes after SARS‐CoV‐2 infection;^33^, ^34^ this is in line with our findings. Previous data from the GRA registry showed that, compared to methotrexate monotherapy, rituximab, sulfasalazine, immunosuppressants (including mycophenolate), and not receiving any DMARD were linked with a greater risk of death. Other csDMARDs/bDMARDs were not linked to death from COVID‐19.^35^ Rituximab attaches to CD‐20 on B‐cell surfaces, depleting this cell type and interfering with antibody production. As a result, B‐cell depletion may impair antiviral immunity, including the production of anti‐SARS‐CoV‐2 antibodies. Of the included patients, 5 received CD‐20 inhibitors (3 patients used it as monotherapy, 1 patient used CD‐20 inhibitors plus antimalarials, and 1 patient used CD‐20 inhibitors plus mycophenolate mofetil). Three of the patients who used CD‐20 inhibitors as monotherapy have died (3 out of the 4 reported mortality cases). Such an increased risk of mortality is a finding that warrants further investigation.

Data from the Centers for Disease Control and Prevention (CDC) and World Health Organization (WHO) documented that 595 309 people in Kuwait were vaccinated during the same period of our study. The Ministry of Health of Kuwait revealed that as of 3 July 2021, 1 452 148 and 923 307 people had received 1 dose and 2 doses of COVID‐19 vaccines, respectively, since the campaign began on 27 December 2020. In the current study, the status of the COVID‐19 vaccine was unknown for most patients (n = 38; 73.08%). Only 2 patients (3.85%) were vaccinated, and 12 patients were not vaccinated. A recent study from the C19‐GRA Vaccine Survey^36^ assessed perception regarding the COVID‐19 vaccine. Of 7005 respondents, 574 respondents (39.4%) reported being unsure or unwilling to receive a vaccine. Almost all of those unsure or unwilling cited worries regarding side effects, safety, and the fast development and deployment of COVID‐19 vaccinations in clinical practice. Despite this, over half of the respondents reported they were pro‐vaccine, while many others expressed varying degrees of apprehension: 98.5% of the unsure respondents and 66.9% of unwilling respondents mentioned that they would be more inclined to get vaccinated if a rheumatologist recommended it, and additional outcomes data are available.^36^ Compared with the general population, systemic IRD patients vaccinated for COVID‐19 showed comparable adverse events.^37^ To boost vaccine efficacy, most patients were willing to temporarily discontinue receiving DMARDs. The low incidence of rheumatoid arthritis flare‐ups necessitating treatment was reassuring (less than 5%).^37^ This underlines the value of developing effective educational initiatives to boost the acceptance of the COVID‐19 vaccine in Kuwait.

The available data highlights the vital relevance of vaccine safety and effectiveness concerns for IRD patients, which have persisted despite widespread vaccination. Educational initiatives aimed at increasing awareness and confidence in vaccines and the potential advantages of vaccination and combating the propagation of misleading information should be designed by Kuwaiti health authorities.

To our knowledge, this is the first study that assesses the sociodemographic characteristics and investigates the factors linked with hospitalization, complications, and death among IRD patients in Kuwait with a confirmed diagnosis of COVID‐19. The main limitation of our study was the relatively small sample size of the included patients, and most of our study participants were from Jaber Alahmed Hospital. Because only individuals with severe symptoms are tested for COVID‐19 in many countries, the C19‐GRA registry has certain drawbacks that include a potential selection bias toward more severe cases. Moreover, the included rheumatologists who reported cases were under marked stress to offer front‐line medical treatment to all COVID‐19 patients; thus, they may have been unable to submit cases or reported them late. We recommend that future clinical trials with larger sample sizes should address the association of different anti‐rheumatic medications with COVID‐19‐related outcomes among IRD patients.

## 5 CONCLUSIONS

Due to the fast gathering of data during the COVID‐19 pandemic, very early characterization and distribution of information about COVID‐19 in patients with IRD have been possible. Moreover, we could examine how sociodemographic and IRD characteristics, therapies used before COVID‐19 diagnosis, and medications were given after diagnosis affect the severity of COVID‐19 outcomes. The current study's findings would provide timely real‐world evidence where considerable gaps in the literature exist, providing physicians with information on the treatment options for IRD patients diagnosed with COVID‐19 and a better knowledge of potential risk factors linked to poor COVID‐19‐related outcomes in the IRD population.

## AUTHOR CONTRIBUTIONS

Fatemah Abutiban wrote the manuscript with input from all authors. All authors reviewed the results and approved the final version of the manuscript.

## CONFLICT OF INTEREST

All authors declare no conflict of interests.

## ETHICAL APPROVAL

The article is approved by The Standing Committee for Coordination of Health and Medical Research – Ministry of Health – State of Kuwait.

## MEDICAL WRITING ASSISTANCE

Medical writing assistance in the preparation of this article was provided by Dr Mahmoud Ebada, Dr Omar M. Hussein, and Dr Reham Elgarhy of RAY‐CRO.

## Supporting information

Supplementary Material
