# Supplementary material for: COVID‐19 outcomes among rheumatic disease patients in Kuwait: Data from the COVID‐19 Global Rheumatology Alliance (C19‐GRA) physician registry
Source: Int J Rheum Dis. 2022 May 11;25(7):743–54. doi: 10.1111/1756-185X.14332 (PMC9347605; doi:10.1111/1756-185X.14332)
Supplement: Supplementary file 1 — Supplementary Material [file APL-25-743-s001.docx]

**SUPPLEMENTARY TABLES**

**Table S1.** Immune modulating medications immediately before COVID-19 onset.

|  | | Frequency | Total | Percentage |
| --- | --- | --- | --- | --- |
| csDMARDs monotherapy | Methotrexate Monotherapy | 10 (19.2%) | 25 | 48.1% |
|  | Antimalarials Monotherapy | 5 (9.6%) |  |  |
|  | Mycophenolate mofetil/mycophenolic acid Monotherapy | 2 (3.8%) |  |  |
|  | Antimalarials and Methotrexate | 1 (1.9%) |  |  |
|  | Antimalarials and Azathioprine/6-MP | 1 (1.9%) |  |  |
|  | Sulfasalazine Monotherapy | 1 (1.9%) |  |  |
|  | Azathioprine/6-MP Monotherapy | 3 (5.8%) |  |  |
|  | Methotrexate and Antimalarials | 1 (1.9%) |  |  |
|  | Mycophenolate mofetil/mycophenolic acid and Antimalarials | 1 (1.9%) |  |  |
| bDMARDs monotherapy | CD-20 inhibitors Monotherapy | 3 (5.8%) | 12 | 23.1% |
|  | IL-6 inhibitors Monotherapy | 4 (7.7%) |  |  |
|  | IL-1 inhibitors Monotherapy | 1 (1.9%) |  |  |
|  | TNF-inhibitors Monotherapy | 1 (1.9%) |  |  |
|  | TNF-inhibitors Monotherapy | 3 (5.8%) |  |  |
| tsDMARDs monotherapy | JAK inhibitors Monotherapy | 2 (3.8%) | 2 | 3.8% |
| csDMARDs plus bDMARDs | CD-20 inhibitors and Antimalarials | 1 (1.9%) | 7 | 13.5% |
|  | CD-20 inhibitors and mycophenolate mofetil/mycophenolic acid | 1 (1.9%) |  |  |
|  | Methotrexate and TNF-inhibitor | 3 (5.8%) |  |  |
|  | IL-6 inhibitors and mycophenolate mofetil / mycophenolic acid | 1 (1.9%) |  |  |
|  | IL-17 inhibitors and Methotrexate | 1 (1.9%) |  |  |
| csDMARDs plus tsDMARDs | | 3 (5.8%) | 3 | 5.8 |
| Azathioprine / 6-MP plus Colchicine | | 1 (1.9%) | 1 | 1.9 |
| None |  | 2 (3.8%) | 2 | 3.8 |

**Table S2.** Laboratory investigation for the included COVID-19 patients.

| Characteristic | Study Cohort |
| --- | --- |
| Anemia (hemoglobin < 9.2 g/dL) | |
| Yes | 8 (15.4%) |
| No | 38 (73.1%) |
| Unknown | 6 (11.5%) |
| D-dimer > ULN | |
| Yes | 14 (26.9%) |
| No | 25 (48.1%) |
| Not assessed | 6 (11.5%) |
| Unknown | 7 (13.5%) |
| Ferritin >2000 ng/mL | |
| Yes | 4 (7.7%) |
| No | 31 (59.6%) |
| Not assessed | 11 (21.2%) |
| Unknown | 6 (11.5%) |
| Fibrinogen < 250 mg/dL | |
| No | 9 (17.3%) |
| Not assessed | 37 (71.2%) |
| Unknown | 6 (11.5%) |
| Leukopenia (WBC < 5,000/mm3) | |
| Yes | 5 (9.6%) |
| No | 40 (76.9%) |
| Unknown | 7 (13.5%) |
| AST OR ALT (SGOT or SGPT) > ULN | |
| Yes | 6 (11.5%) |
| No | 39 (75.0%) |
| Not assessed | 1 (1.9%) |
| Unknown | 6 (11.5%) |
| Absolute Lymphocyte Count < 1,500/mm3 | |
| Yes | 5 (9.6%) |
| No | 40 (76.9%) |
| Unknown | 7 (13.5%) |
| Platelets < 110,000/mm3 | |
| Yes | 4 (7.7%) |
| No | 42 (80.8%) |
| Unknown | 6 (11.5%) |
| Triglyceride >133 mg/dL | |
| Yes | 1 (1.9%) |
| No | 15 (28.8%) |
| Not assessed | 30 (57.7%) |
| Unknown | 6 (11.5%) |
| Splenomegaly or hepatomegaly | |
| Yes | 2 (3.8%) |
| No | 39 (75.0%) |
| Not assessed | 5 (9.6%) |
| Unknown | 6 (11.5%) |

**Table S3.** Descriptive analysis of the days of hospitalization of the rheumatic disease patients diagnosed with COVID-19.

|  | | Duration till symptoms free^†^  (Days of hospitalization) | |
| --- | --- | --- | --- |
|  | | **Mean (SD)** | **Median (range)** |
| Age | ≤65 Years (n= 24) | 9 (±6) | 8 (3 - 30) |
|  | >65 Years (n= 9) | 11 (±8) | 10 (0 - 30) |
| Gender | Female (n= 27) | 10 (±7) | 10 (0 - 30) |
|  | Male (n= 6) | 11 (±6) | 9 (6 - 20) |
| Race/ethnic origin | Non-Arab (n=1) | 6 (±) | 6 (6 - 6) |
|  | Arab (n= 32) | 10 (±7) | 10 (0 - 30) |
| Smoking Status | Former smoker (n= 0) | - | - |
|  | Never smoked (n= 28) | 10 (±7) | 8 (0 - 30) |
|  | Unknown (n= 5) | 12 (±5) | 10 (6 - 20) |
| Glucocorticoids at time of COVID-19 symptom onset | No (n=30) | 10 (±7) | 6 (0 - 30) |
|  | Yes (n= 3) | 10 (±8) | 10 (5 - 20) |
| Methotrexate monotherapy^‡^ | No (n= 23) | 10 (±7) | 8 (3 - 30) |
|  | Yes (n= 8) | 8 (±4) | 9 (0 - 14) |
| csDMARDs^‡^ (Other than Methotrexate | No (n= 24) | 9 (4±) | 9 (0 - 17) |
|  | Yes (n= 7) | 13 (12±) | 7 (5 - 30) |
| Methotrexate plus other csDMARDs^‡^ | No (n= 29) | 10 (±7) | 8 (0 – 30) |
|  | Yes (n= 2) | 7 (±4) | 7 (4 – 10) |
| bDMARDs/tsDMARDs monotherapy^‡^ | No (n= 22) | 10 (±7) | 8 (0 - 30) |
|  | Yes (n= 9) | 9 (±3) | 10 (3 - 14) |
| bDMARDs/tsDMARDs plus Methotrexate^§^ | No (n= 26) | 10 (±7) | 9 (0 – 30) |
|  | Yes (n= 4) | 10 (±5) | 9 (6 – 17) |
| Interstitial lung disease | No (n= 29) | 9 (±6) | 8 (0 - 30) |
|  | Yes (n= 4) | 14 (±11) | 10 (6 - 30) |
| Obstructive lung disease | No (n= 33) | 10 (±7) | 10 (0 - 30) |
|  | Yes (n= 0) | - | - |
| Diabetes | No (n= 20) | 10 (±6) | 8 (3 - 30) |
|  | Yes (n= 13) | 11 (±7) | 10 (0 - 30) |
| Hypertension | No (n= 15) | 10 (±7) | 10 (3 - 30) |
|  | Yes (n= 18) | 10 (±6) | 9 (0 - 30) |
| † *Duration till symptoms-free data missed in 19 patients;* ‡ *missing data in 2 patients;* § *missing data in 3 patients* | | | |

**Figure S1** Ten most common COVID-19 symptoms at onset.

**
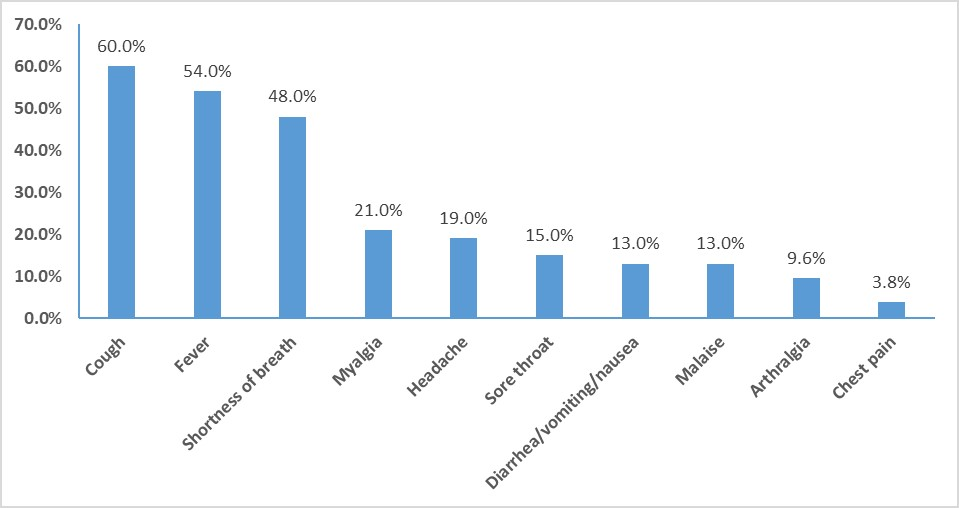
**
